# Individuals who report having benefitted from dialectical behaviour therapy (DBT): a qualitative exploration of processes and experiences at long-term follow-up

**DOI:** 10.1186/s40479-022-00179-9

**Published:** 2022-03-01

**Authors:** Conall Gillespie, Mike Murphy, Mary Kells, Daniel Flynn

**Affiliations:** 1 School of Applied Psychology, University College Cork, Cork, Ireland; 2 Mental Health Services, Cork Kerry Community Healthcare, Health Service Executive, Cork, Ireland

**Keywords:** Borderline personality disorder, Dialectical behaviour therapy, Follow-up, Qualitative research, Recovery

## Abstract

**Background:**

Qualitative research in the area of DBT is limited, particularly at follow-up. The current study explored the follow-up experiences of individuals who previously received a diagnosis of borderline personality disorder and self-report having benefitted from DBT at the time of treatment.

**Methods:**

Individuals who completed 12 months of standard DBT and were a minimum of two years post-completion were recruited. Individual semi-structured interviews were completed with a total of twelve participants.

**Results:**

A thematic analysis generated three main themes which indicated that participants found DBT had a positive impact on their lives in the years after the programme and enabled further development; gave them control over their lives and the ability to manage setbacks and difficult situations; and contributed to healthier and more meaningful relationships with others.

**Conclusions:**

The findings of this study indicated that DBT contributed positively to the participants’ lives and helped advance their recovery in the years after the programme.

Despite the positive impact of DBT, participants required further support in the years following the intervention. Clinical and research implications of these findings are discussed.

## Background

Dialectical behaviour therapy (DBT) is a psychotherapeutic intervention with a growing evidence base in treating individuals who have received a diagnosis of borderline personality disorder (BPD). Over the past three decades, there have been a number of randomised controlled trials across different sites which have demonstrated the efficacy of DBT in treating individuals with this diagnosis (e.g. [1–3]). Results have demonstrated reductions in a number of outcomes associated with BPD including self-harm, suicidal behaviours, and hospital admissions. Similar positive outcomes have also been reported in effectiveness studies where DBT was delivered in community settings [4–6]. International health guidelines including the American Psychiatric Association [7] and the National Institute for Health and Care Excellence [8] recommend DBT as an evidence-based intervention for individuals with a diagnosis of BPD.

Despite the growing evidence base for DBT in treating this presentation, less is known about follow-up outcomes of the treatment. The DBT studies that have explored follow-up outcomes have been quantitative in nature and have typically focused on specific target outcomes of the treatment such as self-harm and suicidality. Overall, the findings of these studies suggest that the DBT treatment effects are maintained up to two years post-intervention [9, 10]. However, little is known about any aspect of DBT beyond this timeframe.

Although these findings are promising, the qualitative long-term follow-up experiences of individuals who have completed DBT have not yet been captured. In general, qualitative DBT studies exploring the experiences of individuals with a diagnosis of BPD are limited. A small number of studies have explored participants’ experiences of DBT, with all studies carried out either while participants were still in the DBT programme, at the end of treatment, or within several months of finishing DBT (e.g. [11–15]). A recent systematic review by Little et al. [16] included seven qualitative studies, with studies focusing on two main areas – 1) Evaluation of the DBT programme components (e.g. the skills group and individual therapy) and 2) The impact of DBT on the participants’ lives. The qualitative research identified key outcomes experienced by participants which were not captured within the research outcomes of quantitative DBT studies including: DBT being perceived as a life changing progression, the development of self-efficacy, and embraced acceptance of difficulties [16]. Synthesis of the qualitative studies indicated that participants placed more emphasis on these outcomes than on outcomes typically reported in quantitative research. Little et al. therefore suggest that the current outcome measures used within clinical practices may not capture all the changes experienced by individuals undertaking DBT, with more qualitative studies needed in order to capture these participant experiences.

Due to the limited qualitative research in the area of DBT to date, the processes through which treatment might be impacting are also less explored. Process research explores possible mechanisms of change through which individuals engaged in therapy can improve [17]. When investigating change mechanisms, both specific and common factors may be considered. Specific processes relate to mechanisms of change which are based in the theory underlying the proposed treatment [18]; common factors include aspects of treatment common to all therapies such as the therapeutic alliance, treatment credibility, and self-efficacy [19]. Based on the synthesis of the available qualitative DBT literature to date, Little et al. [16] suggest a number of potential processes in DBT. These include 1) learning and using new skills; 2) taking responsibility for change; 3) the therapeutic relationship; and 4) increased hope. Other potential processes for individuals receiving treatment for BPD include creating a safe, caring environment where participants feel valued and can actively develop a better understanding of their difficulties, in addition to promoting practical change [20].

The current study aims to address a number of gaps in the DBT literature. Firstly, this study aims to add to the limited qualitative research in the area of DBT. Secondly, it aims to expand our knowledge of the follow-up qualitative experiences of individuals who previously received a diagnosis of BPD and completed standard DBT at least two years previously. Specifically, the study aims to explore the long-term follow-up experiences (positive or negative) of those who self-report having benefitted from DBT at the time of originally undertaking the intervention. The benefits and positive impact of DBT on participants’ lives at post-intervention has been reported in previous qualitative DBT literature. However, to the best of our knowledge, there has been no peer-reviewed research exploring individuals’ experiences at longer term follow-up. This study does not assume that the findings at follow-up will be specifically as a result of DBT but rather aims to explore the individuals’ lives more broadly a number of years after completing the programme, with emphasis on how participants feel DBT has affected their psychological adjustment and recovery. It is hoped that this study can add to the growing DBT literature and inform practice about the potential long-term impact of the treatment. The study aims to answer the following research questions:

1. How do individuals who report having benefitted from DBT report their lives, their coping, and their approaches to problems at long-term follow-up?
2. What, if any, are the DBT-related processes which these participants identify at long-term follow-up as having affected their lives?
3. Do individuals who feel they benefitted from DBT at the time continue to report benefits at two or more years post-completion?

## Methods

### Study setting

The current study took place within a region in the Republic of Ireland. In Ireland, DBT is typically delivered in outpatient community mental health settings [5, 21]. Individuals who are attending their adult mental health services within this region can be referred to one of four DBT teams by a member of their community mental health team. Participants for the current study were recruited from across all four DBT sites. Each team delivered the standard 12-month DBT programme (as outlined in Linehan, [22, 23]) consisting of individual therapy, weekly group skills training, phone coaching, and consultation team meetings for the DBT therapists. Ethical approval for the current study was obtained from University College Cork Clinical Psychology Research Ethics Committee.

### Participants and recruitment

The target population for the present study consisted of a subset of individuals who engaged in DBT - individuals who had completed the standard DBT programme and who retrospectively indicated that they experienced some benefits at the time of originally participating in the intervention were recruited. This target population was selected as the current study wished to explore the long-term follow-up experiences of these individuals. At the time of DBT participation, all individuals were engaged with adult community mental health services. In Ireland, these services provide treatment to adults who are experiencing moderate to severe mental health difficulties. All individuals had received a diagnosis of BPD by the treating consultant psychiatrist on the team and were experiencing severe emotional and behavioural dysregulation prior to commencing DBT. Individuals who were recruited for this study were still in contact with local community mental health services in some capacity.

Inclusion criteria for participants were:

- Individuals who had completed a standard DBT programme. Typically, individuals complete a 12-month programme, however, on occasion, some individuals may complete a 6-month programme. This applied to one participant in this study. Upon completing the DBT programme, individuals typically cease individual therapy with their DBT therapist. A number of ‘booster’ individual sessions may be offered to individuals in the months after completing DBT or in some cases, a formal DBT extension may be agreed.
- Individuals who were a minimum of two years post-completion of DBT at time of recruitment.

Exclusion criteria were:

- Individuals currently participating in a DBT programme.
- Individuals who had completed DBT less than two years ago.
- Individuals who were deemed to be intoxicated as a result of drugs or alcohol at the time of the interview.
- Individuals who were overtly emotionally dysregulated or appeared to be in a dissociative state or psychotic state at the time of interview.
- Individuals who indicated that they did not find any benefits to DBT at the time of originally undertaking the DBT intervention.

Recruitment took place between March 2020 and November 2020. When considering sample size, it was hoped to recruit a sufficient number of participants to achieve data saturation. Saturation occurs when no new codes or themes can be generated from the data, thus positively impacting a study’s quality and content validity [24]. There is no predetermined number of interviews which guarantees saturation, as this can depend on the type of study design and quality of the data [24]. When designing the current study, a number of considerations were made with regard to potential sample size. Firstly, the study was interested in a subset of individuals who engaged with DBT, therefore it was anticipated that it would be a relatively homogenous sample. Secondly, research around data saturation in qualitative research has indicated that saturation can often be achieved within the first 10 to 16 interviews (e.g. [25, 26]). Therefore, the above considerations provided a rough guide when designing this study as to how many participants may be required to achieve saturation.

The DBT teams in the region were orientated to the study by the authors and received study information leaflets and fliers. The DBT therapists were then invited to share this information with their respective community mental health teams. Both the DBT therapists and other community mental health team members then shared this information with clients of the service with whom they had contact with in the course of their work. Individuals who met the inclusion criteria were given a study information leaflet which outlined the purpose of the study and contained the first and third authors’ contact details. If individuals felt that they had benefitted from DBT in some capacity at the time of treatment, they were invited to participate by contacting one of the authors. Informed consent was obtained from all participants prior to participation.

### Sample characteristics

A total of thirteen individuals expressed interest in participating in the study. One individual ultimately did not take part as they did not wish to complete the interview online or by telephone, resulting in a sample size of twelve participants. The sample was predominantly female (75%) and ranged in age from 25 to 73 years old (*M* = 46.08 years, *SD* = 15.06). The majority of participants were either married or in a relationship (58%), while half of the participants (50%) were employed at the time of interview. Most participants (92%) were taking medication for their mental health difficulties. Participants had completed DBT between 2011 and 2018 and the mean number of years since completion was 4.42 years (*SD* = 2.43). A quarter of participants (25%) had experienced at least one hospital admission in relation to their mental health difficulties following the completion of DBT (mean number of admissions among these participants = 2.3).

### Data collection

Individual semi-structured interviews were conducted between August 2020 and November 2020 by the first author. Initially, it was planned to complete face-to-face interviews. However, as a result of the COVID-19 pandemic and consequent University guidelines, interviews were completed by either telephone or online video calls using Google Meet. The length of the interviews ranged from 37 to 71 min long (*M* = 50.05 min). The interviews were guided by an interview schedule which was developed by the authors. The questions were informed by previous DBT literature and were phrased in an open and broad manner in order to explore participants’ lives before DBT, the experience of DBT, and how participants would describe their lives now. This broad approach has been used in previous qualitative DBT literature exploring participants’ experiences of the programme. A similar approach was adopted in the current study to enable exploration of experiences over the years since completing DBT. Additional questions in the interview schedule aimed to broadly tap into processes that have been highlighted in previous DBT qualitative literature including self-efficacy, use of skills, hope and improved relationships. It was also hoped that the broad and open questions might help identify additional outcomes and processes not highlighted in previous research.

### Data analysis

All interviews were transcribed and analysed by the first author using thematic analysis, which is a method for identifying, analysing, and reporting patterns or themes within data [27–30]. This method of analysis was chosen due to the exploratory nature of the research questions. Braun and Clarke’s six phase approach to analysis was followed which began by the author familiarising himself with the data through the transcription process, re-listening to the audio recordings, reading the transcripts a number of times, and making notes. The data was then analysed systematically by coding, identifying and providing labels with regards to relevance to the research question [28]. When the coding was completed, themes were then generated, reviewed and defined in an iterative process before writing up the results. In order to cross-check the analysis process and ensure a shared understanding of the data, both the initial codes and the generated themes were reviewed and discussed with the second author.

With regards to epistemological position, the research was approached from an essentialist perspective, where there is an assumed reality in terms of the experiences reported in the data [28]. A combination of both bottom-up (inductive) and top-down (deductive) approaches were used during the data coding and analysis..

### Reflexivity

When completing the interview and analysis process, the first author reflected on his personal preconceptions and experiences that might shape the research process. The reflexivity process was iterative and a reflective journal was kept throughout. The first author was involved in previous DBT research and had a good understanding of the intervention. Therefore, his existing knowledge in the area may have resulted in researcher bias and thus influenced his expectations about participants in the study. The second author, who reviewed the codes and themes had no prior experience in the area of DBT but was experienced in qualitative research methods. This allowed for different perspectives to be brought into the analysis process and therefore increase the validity of the findings [31].

## Results

**Fig. 1:**
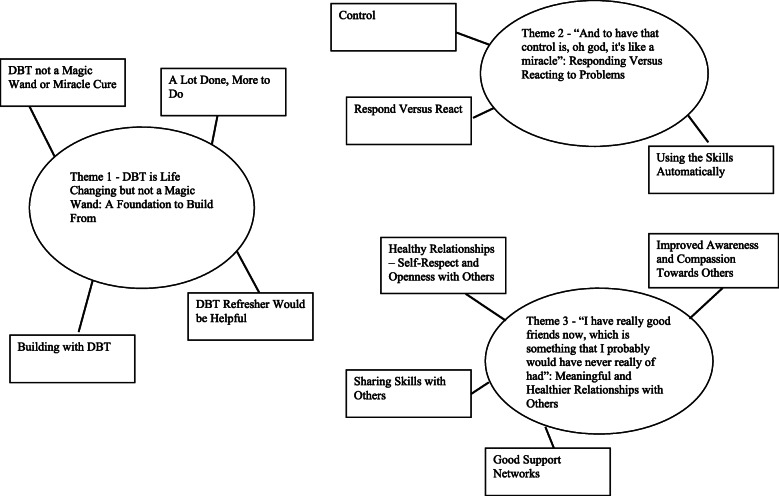
Thematic Map

A total of three superordinate themes were generated from the data: 1) DBT is Life Changing but not a Magic Wand: A Foundation to Build From; 2) “And to have that control is, oh god, it’s like a miracle”: Responding Versus Reacting to Problems; and 3) “I have really good friends now, which is something that I probably would have never really of had”: Meaningful and Healthier Relationships with Others. A thematic map of the superordinate and subthemes is outlined in Fig. 1.

### Theme 1 - DBT is life changing but not a magic wand: a foundation to build from

#### DBT not a magic wand or miracle cure

Participants reflected on the life changing impact DBT had on their lives. However, for many participants, completion of the DBT programme did not mark the end of their therapeutic journey but rather a starting point for further development. Some participants explained that prior to starting the programme, DBT had been pitched by referral agents as almost like a panacea or a “miracle cure” but that this was not the case. However, although it was not the “be all and end all”, it did provide participants with a life changing experience from which to build on. As one participant described:

“But I mean, you know, DBT, it's not the magic wand but I have to say, I wouldn't be here without the course. I really wouldn't like, do you know.” (P3)

Another participant spoke about how they became aware after completing the programme that “this isn’t the end of it”, while another participant acknowledged that DBT required continuous effort even after completing the programme:

“But it’s, DBT is not a miracle cure, it’s something that you have to work at” (P11)

Although participants highlighted the positive impact of DBT on their lives, there were still issues to be explored and it was clear that further support was still required after completing the programme. As one participant noted:

“Yeah so it's kind of been, my life has definitely changed an awful lot since DBT. I would say, definitely for the better. DBT really did change my life … Like obviously DBT was great and it helped me learn loads but I still had problems after it.” (P4)

#### A lot done, more to do

Participants described engaging in a wide variety of supports in the years after completing DBT. For example, some participants continued to engage in work with their DBT Individual Therapist for a period of time after completing the programme. This included a number of ‘booster sessions’, which are offered to participants in the months following programme completion, or a formal extension in the 12 months post-completion. A small number of participants also described engaging with the psychologist on their community mental health team, who had also been their DBT Individual Therapist, for psychological intervention around other issues at times during follow-up. Participants noted finding this work beneficial, both in terms of the therapeutic relationship and connection they had with their therapist, and consolidating the skills learned on the programme:

“Think I definitely needed the extension. Like I think it hammered home the last bit of the skills and the bit of, the building blocks of whatever was going on in my head.” (P1)

Similarly, another participant noted:

“I have been so privileged and I honestly believe that having (DBT Therapist) as a follow-up to DBT, I have been very fortunate. Because it is very easy to forget about certain aspects or forget to use your skills” (P8)

Following completion of DBT, many respondents continued to engage with mental health supports and services including trauma services, peer groups, and further individual therapy. Some participants also took part in a post-DBT coaching programme called GLOW – Goals for Life: Opting for Wellness [32]. With regards engagement with their mental health service, some participants noted that it was difficult to find further therapeutic support within the service once they finished DBT due to them no longer being in crisis. In some cases, their only engagement with their service was brief outpatient medical review appointments every few months. Some participants noted their frustration with this, for example, one participant described:

“Like you know, I feel like from the psychiatrist's point of view, I've done DBT now so that's as far as they can, that's all they can offer me” (P2)

With regards to peer groups, although some participants did have the opportunity to engage in such groups in the years following the programme, the difficulties associated with sustaining such groups were also highlighted:

“I did some support groups in the years after but you know, difficult, you know, if people are going to attend or not, that kind of stuff” (P6)

#### Building with DBT

Where participants did go on to receive further therapeutic supports, it was clear that they felt DBT had given them a strong foundation which helped facilitate this further development. As one participant noted,

“I would say that as a certainty, I would say that DBT was a beginning point. So a start point.” (P6)

For example, several participants spoke about how they were able to use the skills when completing future trauma work that could not be done in DBT (DBT Prolonged Exposure [33], a protocol developed specifically for treating trauma, was not available in Ireland pre-2018). Some participants went on to engage in other therapeutic work such as schema therapy and highlighted how the skills learned in the DBT programme benefitted them while engaging in the work:

“Because you had the skills, DBT is so good for, I suppose what it is built for is, just bringing you back to a kind of baseline from which you can then work. And I think having had that foundation, being able to delve into schema was much easier. And much more beneficial because of the stabilising effect of DBT” (P12)

In addition to further development of self, participants also spoke of how the skills learned in DBT enabled them to develop professionally in terms of furthering their education or careers. A number of participants went on to pursue academic courses in the years following DBT and spoke about how the skills have helped them:

“So, I think they’ve, the skills I’ve learned have helped. If I didn’t have the skills, I don’t think I would’ve been able to go into the course” (P1)

Similarly, participants spoke about how this helped facilitate their return to work:

“So yes there were positives, it gave me a confidence, I went off and did that course, I did another course, you know, a course about getting back to work and stuff like that” (P3)

#### DBT refresher would be helpful

Finally, almost all participants expressed the wish to see some form of a brief DBT refresher or follow-up take place. Although there was a DBT Skills Review Group available in some services within the region in which this study took place, some participants indicated that this was not well attended. Although almost all participants indicated that they would like some form of a refresher, suggestions on what this might consist of and how it could be delivered varied. Participants acknowledged the possible logistical difficulties of a follow-up refresher, with one participant suggesting it could even take place online. Other participants proposed that a follow-up check-in become a mandatory part of the programme. Some suggested how between one to four group check-ins would be helpful to see how people are getting on and share follow-up experiences:

“Because you have invested, and the tutor has invested so much time and energy, and you have as well, and it would be nice to catch up with your group and see, you know, have things changed for you. Or someone might come up with, oh, I used this skill or that skill. Those things like that and I think it would give you more, em, confidence I think in yourself as well if you are using it.” (P8)

Other participants noted that a refresher would be helpful to “brush up” on aspects of the skills that they may have forgotten and to ensure they are still using skills correctly:

“And those are the pieces that I think, would be very nice to get a refresher course on. You know, just to kind of, I suppose it's like everything, if you learn a new skill and if you're only using half of it, well you are going to forget the other half” (P5)

While most participants expressed their wish to see some form of a refresher group occur, two participants had a contrary view noting their preference for a refresher with an individual DBT therapist as opposed to with a group.

### Theme 2: “And to have that control is, oh god, it’s like a miracle”: responding versus reacting to problems

#### Control

The theme of participants now having control of their lives was evident across the interviews, with participants highlighting how the programme gave them skills to cope with setbacks and difficult situations. Several participants reflected on the differences between how they would cope with problems in the years prior to completing the programme, where crises would often lead to hospitalisations, and how they would now. For example, one participant noted how hospitalisation was a default way of coping for them for decades prior to DBT:

“Because in the early days I would be admitted there for crisis intervention at the drop of a hat. And nobody ever sort of sat down with me and said, "Oh you should try and cope". If I couldn't cope I would just go running up to that psychiatric hospital. You know, it could be in the middle of the night or sort of 3 in the morning. And they would admit me and would discuss the paperwork later. Normally it involved a sort of increase in medication and long stay until you felt ready to go home.” (P9)

Similarly, another participant spoke about how they were “continually in and out of hospital” prior to DBT and how that has changed since:

“No you were always hospitalised. Whereas now, I am not being hospitalised” (P7)

Participants reflected on their experiences of frequent hospitalisations in the years preceding DBT and it was clear that they now felt a greater sense of control over their lives:

“And I suppose I would kind of feel that my life is, to some extent anyway, within my own capacity to deal with” (P10)

Participants described how they had experienced a number of setbacks in the years after DBT, such as serious medical diagnoses or the death of a loved one and had been able to use their skills to effectively cope with them. For example, one participant described their experience of receiving a life changing medical diagnosis a number of years after completing the programme. They spoke about how in the past, they would have been devastated and seen it as a personal punishment, whereas now they were able to manage it effectively:

“The following year then I found out I had (medical diagnosis) … But the fact I didn't have a big breakdown when I was told … Em, I got on with it, I got on with things, do you know, I didn't need to ring the doctors, I didn't need to ring the nurse to say I was upset or anything, I just told them at our next meeting and I, I took it very well (laugh)” (P11)

Another participant spoke about experiencing a setback and attempting suicide but used their DBT skills to pull them back and seek appropriate support:

“And then just in the back of my head I was like, you know, what am I doing? And then I was like, I am not thinking clearly. And then I tried to use the skills to kind of pull me back and thankfully it did … Do you know what I mean, like before the DBT, my self-harming and the suicidal tendencies and all that, I would have to be hospitalised, do you know what I mean? Whereas now, okay even though it came so close last year, I know that if I didn't have the skills, I actually genuinely mean this from the bottom of my heart, I wouldn't be here today if I didn't have the skills from the DBT programme, do you know.” (P3)

Some participants also spoke about how the skills have helped them “bounce back” quicker from setbacks in the years after DBT. For example, one participant noted:

“I took a bad turn there about twelve months ago but even at that, when I was down at my lowest, I was still using the skills from DBT. And I found that, well what I found with the DBT, you might have a bad day before, and the bad day meant I'm not fucking even getting out of bed like, you know. Whereas with the DBT and stuff, it might only be a couple of hours” (P5)

While almost all participants spoke about how DBT helped them to manage various setbacks in the years after the programme, a small number of participants spoke about how experiencing setbacks made them feel like they were “a failure at DBT” or that “all the hard work was gone down the drain”. However, these participants spoke about how they were able to remind themselves of their progress, challenge their negative thinking, and re-engage with the skills.

In addition to being able to manage major setbacks in their lives, participants also spoke about how they are better able to manage difficult emotions and situations in the years after completing the programme:

“I feel, em, an ability to self-soothe and mind myself, which I think is all I ever really needed. And everything else can happen or not happen … Yeah, it's something I was never able to do. It's something I always, I used to drink, I used to find boys, I used to do anything else” (P12)

Participants also expressed their confidence in managing future difficulties by using the DBT skills:

“I mean I have had no hospitalisations since. For the last two years. And I don't intend to be, because I use my skills to, if I ever feel, I mean I do get my days and there might be days where I feel, you know, but I am able to stop it. I am able to hold it in its tracks” (P8)

#### Responding versus reacting

Participants identified how being able to respond rather than react to difficult situations has helped them to cope with problems in their lives now. Many participants spoke about how they now stop and take a step back before responding to these situations rather than reacting impulsively, which has allowed them to manage the situation more effectively and prevent the situation from getting worse. As one participant noted:

“Because I do find that the times I do act on my impulses, usually aren't good. Without thinking. So I suppose, a lot of what I learned from DBT is to not act as much, take a step back and think for a second.” (P4)

Similarly, another participant said:

“I step back first. Before I would have gone into my own little shell or bubble, and just stayed there. And like I might have gone head first into situations not knowing what was going on. But now I will step back first, breath, and then if necessary, go head first into the situation (laughs). But I definitely step back first” (P1)

Participants also spoke about how they were able to respond rather than react to situations by stopping themselves getting to the point where they would usually become dysregulated:

“I suppose there is a lot of things you know how to do, but in the spur of the moment you kind of can’t, sometimes you kind of go from 0 to 60 in milliseconds. But it's trying to catch yourself before you get to 60 and that's what DBT kind of helped with.” (P2)

#### Using the skills automatically

Almost all participants commented on how they now use the skills as part of their typical coping repertoire. For example, one participant explained:

“You are using the tools without knowing you are doing them, you know, most of the time … if you read all the files again you realise, ‘Oh god I am doing those everyday’” (P11)

Some participants spoke about how they cannot remember most of the names of the skills but have generalised them to the extent that they have become a natural part of their everyday behaviour where they “just do it automatically”, and they no longer need to a make a mindful choice to select them:

“And I find even to this day with the stuff from the DBT and things, it's when you sit down at the end of the day and you recall your day. You can see the parts you have used … That they automatically just become part of you.” (P5)

### Theme 3 - “I have really good friends now, which is something that I probably would have never really have had”: meaningful and healthier relationships with others

The final theme generated from the data relates to the connection that participants described with other people in the years following DBT. This theme was evident throughout the interviews across participants.

#### Healthy relationships – self-respect and openness with others

Prior to DBT, participants reflected on how their relationships with others were impacted as a result of their difficulties, with some participants describing how they had lost touch with many of their friends. Participants spoke about how their relationships have improved as a result of DBT and how they have continued to improve in the years since completing the programme, where they now experience more positive and open relationships with others and have been able to reconnect with old friends:

“Em, friends, I have gone back to. I mean my anxiety was, I couldn't go into town on my own or anything like that. And I would avoid my friends, I would avoid their calls, I would avoid everything. So now, they all know what's wrong with me and, you know, I am still in contact with all of them.” (P8)

Another contributor to participants’ healthy and more meaningful relationships in the years following DBT was their openness about their difficulties with others. Many participants spoke about how their openness about their difficulties with other people has had a marked impact on their lives and has led to a deeper connection with others:

“Talking to people and you would be surprised how many people that I now know, that are opening up to me about their own problems, because I have opened up to them and I have nothing to hide. And if I see them now, I get on so much better with people than I ever did because I am hiding nothing, they are hiding nothing, and em, you don't feel quite so isolated.” (P9)

Some participants also described how they tended to remain in negative peer relationships prior to DBT. However, in the years following the programme, an important aspect that contributed to healthier relationships was their ability to pull-back or eliminate negative relationships in their lives:

“So when I was with DBT, I would have had a lot of crappy friendships. And people would have used me or just kind of, when there was no one else around they would take me or whatever. And I kind of got rid of all of that.” (P4)

Participants also described how they now engage in more self-compassion and put their own mental well-being first when considering certain relationships. For example, one participant spoke about how they are in the process of pulling back from a friend of many years who has a tendency to be direct and hurtful towards others. Even though the participant knew it would be difficult, and that they would feel bad about it, they realised the effects of this relationship on their own mental health:

“I'm actually, something I wouldn't have done before DBT, I am thinking of myself and what's best for me and my whole family … The whole time gently pulling back because I think it will be healthier for me.” (P11)

#### Improved awareness and compassion towards others

Related to this improved connection was participants’ improved understanding and compassion towards others, where they described being more aware of other peoples’ difficulties and how they respond to them. For example, one participant explained:

“Yeah, I just I, some people think I make excuses for people now, you know, when someone else is cross or angry and I’m just like, ‘Leave them alone, you just don’t know what’s going on in their head’” (P11)

Another participant described the experience of being in an argument with others and how they respond differently now than how they would have before:

“And if you're listening to it, like you don't have to be a rocket scientist, but if you listen to it and you know people, you can put the truth together to what they are actually angry about. And then it doesn't bother you. What ends up happening then is, you end up in a scenario where you want to help them.” (P5)

#### Sharing skills with others

Through the improved understanding and compassion towards other people, participants also described how they have been able to help others in the years after DBT. Participants spoke about teaching the skills to others, either individually or when engaging in a peer group:

“But even like, say with friends, I am able to calm them down as well. Calm them down by giving them, saying to them do such and such a thing for a few seconds, do such and such a breathing exercise do you know what I mean? It helps. They would say, ‘Where did you learn that?’” (P1)

Participants also spoke about how being able to help others has also had benefits for them. By teaching the skills to others, participants have been reminded of skills they may have forgotten in the years since DBT. Being able to help others using the skills also provides a rewarding experience for participants:

“I was, like the one thing the DBT has allowed me to do is, in the last two years, I have been able to help three or four people that have suffered panic attacks, anxiety, and the whole lot … You know, and I suppose for me, that was the reward for me.” (P5)

#### Good support networks

Finally, participants noted that they have strong support networks in their lives now which includes both family and friends. It was clear that these networks have been invaluable sources of support for participants in the years since finishing DBT. For example, one participant described:

“Even if it’s something really, really trivial I will discuss it because, you know, it’s that old saying a problem shared. So I talk more freely and even if it’s not my wife, I will talk to a friend. You know, most of my close friends know my situation so you know, if something is bothering me, I’ll tell the closest person I’m to at the time and you don’t feel quite so isolated” (P9)

The majority of participants also noted that they have continued to stay in contact with people from their DBT group in the years after the programme, with some going on to become close friends:

“So that group they really changed my life. Those people were inspirational beyond inspirational for me … Like I still talk to some of them.” (P4)

## Discussion

The current study aimed to add to the limited qualitative DBT literature to date by exploring the long-term follow-up experiences of individuals who have completed DBT and found it to be of benefit at the time. Specifically, the study aimed to explore how these individuals reported their lives and their coping a number of years later, whether they continue to report benefits from DBT, and the potential processes at long-term follow-up. A thematic analysis of the participants’ follow-up experiences generated three main themes, which indicated that participants found DBT had a positive impact on their lives and enabled further development, gave them control over their lives and their ability to manage setbacks and difficulties, and contributed to the formation of meaningful connections and relationships with others. Overall, participants indicated that DBT contributed positively to their lives and advancing their recovery in the years following the programme.

Participants reflected on how DBT had had a life changing impact on them, which is similar to findings from previous qualitative DBT research [16]. The findings from this study indicated that participants continued to acknowledge this impact a number of years later. However, it was evident from the first theme that the completion of DBT was not the end of participants’ therapeutic journey but rather a building block for further self-development and ongoing recovery. Participants identified the transformative effect DBT had on their lives, while also highlighting that it was not a miracle cure. Participants identified how the skills learned from the programme enabled them to engage in further therapeutic supports or to pursue further education and careers. One way of considering these findings may be from the perspective of the broaden and build theory of positive emotions [34], where participant’s ability to effectively manage their emotions allowed them to build further psychological resilience, personal resources, and an upward path of further development.

The concept of DBT being a building block for further development rather than a miracle cure is something that could be considered within services, as there may be a misconception among non-DBT clinicians that individuals will not require further support after completion of standard DBT. One participant in this study described how DBT was portrayed by the referral agent on the multidisciplinary team in their service as the only treatment which could help her and that it would solve all her problems, reflecting other participants’ experiences in previous DBT literature [13]. However, quantitative DBT studies that report follow-up data have indicated that the majority of participants (51 to 78% where reported) were engaged in some form of therapeutic support between 12 and 30 months post-DBT [35]. This may be expected considering the chronic nature of participants’ difficulties prior to DBT, and the need to complete further work in areas which may not have been achieved in the first 12 months of intervention given the focus on behaviour stabilisation.

Some participants also highlighted the lack of appropriate supports within mental health services for individuals who have completed DBT and are no longer in crisis but perceive that they require further therapeutic support. Getting less from the treatment system following progress in therapy is understandable, but raises important questions about contingency management. Ways of meeting these individuals’ needs, and considering how treatment systems can further reward people for progress, may be something to consider for services going forward. It is worth considering that there were four stages of DBT originally conceptualised by Linehan [22] but public health services generally offer only stages one (behavioural stabilisation) and two (treating trauma). Therefore, the DBT model does not guide these services on what to offer after stage one and two. Stages three and four of DBT as conceptualised by Linehan includes addressing general happiness and dealing with the problems of everyday life, and finding a deeper spiritual existence and capacity for joy. With this is mind, a broader implication of the findings relates to the need for mental health services to address the goals such as those highlighted in stages three and four of DBT in order to continue to help clients with their recovery process.

The majority of participants also expressed their desire for some form of a DBT refresher or follow-up in the months or years after DBT. For example, a number of participants suggested a group reunion in the months after the programme to revise skills and to share and learn from others’ experiences since finishing. Although there are monthly DBT Skills review groups in some areas in the region where this study was completed, which are open to all individuals who completed the programme, they are not currently delivered in others at present. In the areas where there are DBT Skills review groups, some participants felt that these were too theoretical and suggested a more flexible and experiential approach. The functions of these DBT Skills review groups are skills strengthening, skill generalisation, and enhancing motivation. However, despite attempts over the years from services in this study to develop an effective format for this group based on feedback from service users, attendance at these groups has been limited. DBT Skills review/graduate groups are delivered in many areas internationally, however research in this area is still very limited. One pilot study conducted in the United States identified the potential benefits of these groups [36], with future research required to further understand DBT aftercare programmes and how to optimise them.

Participants highlighted the autonomy they now have over their lives in the years since completing DBT compared to previously, where they are able to effectively manage setbacks and difficult emotions and situations in their lives. These findings are similar to the experiences described in previous DBT literature when participants were either still in DBT, at the end of the programme, or several months after completing the programme [11, 14, 15]. Participants directly attributed the skills to helping them manage these situations and noted how these skills have now become an integral part of their everyday lives and behaviour repertoire, which is similar to previous findings [37]. The acquisition, strengthening and generalisation of skills has been highlighted as an integral process of DBT [38] and these findings suggest that participants have consolidated these skills in the years following completion. The impact of developing self-efficacy, as described by participants in this study, reflects findings from both previous DBT literature and research exploring recovery in individuals with a diagnosis of BPD [16, 20].

Participants reported strong and meaningful connections with others in the years following DBT. Similar improvements in relationships have been reported in previous DBT research [11, 14]. In addition to learning new interpersonal skills, this may also be as a result of increased awareness and acceptance [16]. The current study identified similar findings, where participants described now having increased relationship mindfulness. Participants also described how they are more open with others about their mental health difficulties, which has resulted in improved relationships. These may have contributed to participants now having strong support networks, which they described as being an important source of support in their lives at present. This finding regarding improved relationships makes sense within the context of DBT skills training modules, and is likely the product of improved relationship mindfulness, emotion regulation, and interpersonal skills respectively. Participants’ improved relationships with others could also be considered through a social learning theory perspective [39] as participants frequently highlighted the importance of the therapeutic relationship with their DBT Individual Therapist, as well as learning from others in the group. Improvements in relationships have also been reported by participants receiving other treatments for BPD, where it has been suggested that the strong therapeutic relationship might provide individuals with the opportunity to develop more secure attachments through experiencing and practicing new ways of relating to others [20].

When considering the processes involved at long-term follow-up, a number of factors may be considered. Based on previous qualitative DBT literature, Little et al. [16] suggested learning and using new skills, taking responsibility for change, and the therapeutic relationship as processes of change. Similar conclusions may be drawn from the findings of the current study. The consolidation and regular use of DBT skills, and the further development of new skills following DBT appear to play an integral role. A small number of participants engaged with their individual DBT therapist for further psychological work in the months after DBT and highlighted the importance and connection achieved through that therapeutic relationship. This additional contact with therapists may be considered from the Vygotskian ideas of scaffolding and the zone of proximal development [40, 41]. Through scaffolding, therapists continue to help participants develop emotional resilience and stretch their development, while gradually reducing support as participants acquire the skills and begin using them independently. Finally, participants’ improved relationships and strong support networks may also contribute to change during follow-up. Katsakou and Pistrang’s [20] meta-synthesis of qualitative studies exploring clients’ experiences of treatment and recovery in BPD indicated that the processes of change are unclear. However, based on their findings, they suggested that treatment characteristics that may facilitate change and recovery include creating a safe, caring environment in addition to promoting practical change; which reflects the proposed mechanisms of change in specialist interventions for BPD. It could be said that DBT in particular is well placed in this regard with its focus on both acceptance and change strategies. However, Katsakou and Pistrang highlight that these suggested processes appear generic, with future research required to identify more specific processes within specialised interventions and additional processes for recovery that occur in conjunction with these. Based on the findings of this study, one potential process specific to DBT may be the generalisation of the DBT skills themselves, which target key areas relevant to this presentation e.g. distress tolerance, emotion regulation and interpersonal effectiveness. Through generalisation of the skills, individuals may develop the ability to coach and guide themselves in effective skill use within a variety of contexts [42]. Participants in this study specifically spoke about how the DBT skills have aided with their recovery in the years following DBT.

In comparison to quantitative research, qualitative research usually consists of smaller sample sizes. Due to these small sample sizes, the issue of data saturation is an important consideration when determining the validity of qualitative research findings. A strength of this qualitative study was that the sample size of twelve participants enabled data saturation to be achieved, where no new codes could be generated from the data, thus improving the quality and content validity of the research. Another strength of the study was recruiting participants from across four different treatment sites to reduce bias. Finally, care was taken to ensure the study methodology was clearly described, as this was identified as a limitation of previous qualitative DBT literature [16].

A limitation of this study was that all participants recruited were still in contact with services in some capacity. As consent to contact individuals for follow-up was not obtained while individuals were participating in their initial programme, individuals who were no longer in contact with services could not be contacted. Although many individuals continue to be involved with services post-DBT in some capacity, there may be a smaller subsection of participants whose experiences may not be accounted for. A consideration for future research may be obtaining consent during the DBT programme to contact participants for potential follow-up research in the future. Such research may allow for a further understanding of the long-term follow-up experiences of those who complete DBT. Another limitation of this study was that it did not include participants who either dropped out of DBT, or completed the programme and did not find it beneficial. Future research may explore these individuals’ experiences which may provide important insight when considering treatment delivery. Another limitation of this research was that many participants went on to engage in further therapeutic interventions following DBT, which may contribute to the positive experiences reported at follow-up. Such a limitation has been highlighted in quantitative DBT studies that report follow-up data (e.g. [2, 9, 10]). This qualitative study found that participants specifically identified the positive impact DBT has had on their lives at follow-up, however, the benefit of further therapeutic supports in conjunction with DBT must also be considered. Finally, due to the small sample size of the present study, the issue of generalisability regarding the findings must also be considered. Future research exploring qualitative follow-up of DBT participants will help determine whether the themes may be generalised across other groups, contexts and settings.

## Conclusion

The current study explored the long-term follow-up processes and experiences of individuals who previously received a diagnosis of BPD and completed DBT. Findings indicated that participants continued to report experiencing benefits from DBT a number of years after completing the programme. Thematic analysis suggested that participants found DBT to be a life changing experience that enabled further self-development in the years following the programme, gave participants a sense of control in their lives and skills to manage difficulties, and contributed to improved relationships and connections with others. Future qualitative research exploring the experiences of individuals who dropped out of DBT, or those who report not benefiting from the treatment, will help further understand follow-up experiences and inform service delivery.

## Data Availability

The datasets analysed during the current study are not publicly available but are available from the corresponding author on reasonable request.

## Abbreviations

BPD: Borderline personality disorder
DBT: Dialectical behaviour therapy
